# Real-World Experiences with VMAT2 Inhibitors in Pediatric Hyperkinetic Movement Disorders

**DOI:** 10.5334/tohm.1023

**Published:** 2025-06-16

**Authors:** Sujal Manohar, Jennifer Jacobe, Rebecca Berger, Joseph Jankovic, Mariam Hull

**Affiliations:** 1 Pediatric Movement Disorders Clinic, Blue Bird Circle Clinic for Pediatric Neurology, Section of Pediatric Neurology and Developmental Neuroscience, Texas Children’s Hospital|Baylor College of Medicine, Houston, Texas, USA; 2 Parkinson’s Disease Center and Movement Disorders Clinic, Department of Neurology, Baylor College of Medicine, Houston, TX, USA

**Keywords:** VMAT2 inhibitors, tetrabenazine, valbenazine, deutetrabenazine, pediatric hyperkinetic movement disorders

## Abstract

**Background:**

Vesicular monoamine transporter 2 (VMAT2) inhibitors are often prescribed for the treatment of hyperkinetic movement disorders such as tics, stereotypy, tardive dyskinesia and chorea. These dopamine depleters have been FDA approved in adults for the treatment of chorea in Huntington’s disease and tardive dyskinesia. Use of VMAT2 inhibitors in pediatric hyperkinetic movement disorders, however, is limited due to lack of pediatric FDA approval. We review the real-world prescribing practices and patient experiences with VMAT2 inhibitors in children.

**Methods:**

We performed a retrospective chart review of patients treated with VMAT2 inhibitors at a pediatric movement disorders clinic from 2011 to 2023. Demographics, indication, medical history, and clinical notes were reviewed.

**Results:**

We identified 340 pediatric patients (65.3% male, average age 11.9 years) who had been prescribed a VMAT2 inhibitor for a variety of hyperkinetic movement disorders (359 total prescriptions) at our large pediatric movement disorders center. Of the 359 prescriptions for VMAT2 inhibitors, 94% included tetrabenazine, 4.6% deutetrabenazine, and 1.4% valbenazine. Most common clinical indication was tics (73.5%), followed by chorea (9.1%) and self-injurious stereotypy (7.1%). Of these prescriptions, 75.8% (N = 275) successfully commenced treatment. Most patients (62.8%) had clinical improvement with an average Clinical Global Impression-Improvement of 1.8 (±1.2), indicating “very much” or “much” improved, but 11.5% experienced no improvement in symptoms. Most patients (63.9%) reported some side effects, most commonly drowsiness; however, only 35 (10.1%) necessitated discontinuation due to side effects. Seven patients were transitioned from tetrabenazine to deutetrabenazine with resolution of side effects, and one to valbenazine with similar effect. Of the 359 prescriptions, 194 (54%) experienced at least one denial from insurance companies; 24.2% were unable to initiate treatment due to barriers such as insurance denials and 19.6% expressed financial concerns regarding medication affordability.

**Discussion:**

Our study suggests that VMAT2 inhibitors are effective for treating pediatric hyperkinetic movement disorders. Furthermore, this study provides insights into barriers to access to these drugs by pediatric patients.

Vesicular monoamine transporter 2 (VMAT2) inhibitors act on the central nervous system and depletes presynaptic dopamine [1,2]. They are commonly prescribed to treat hyperkinetic movement disorders [3,4] including tics, chorea, and stereotypies. These medications do not cause tardive dyskinesia, and are the preferred treatment for chorea and tardive dyskinesia [5,6].

Tetrabenazine, the first selective VMAT2 inhibitor, was approved in 2008 by the Food and Drug Administration (FDA) to treat chorea associated with Huntington’s disease (HD) after showing benefits in a randomized controlled trial (RCT) [7]. In 2017, deutetrabenazine was FDA approved to treat tardive dyskinesia and chorea in HD while valbenazine was approved to treat tardive dyskinesia this same year, and subsequently approved for HD chorea in 2023 [8,9]. In addition to their FDA-approved indications, all three VMAT2 inhibitors are used off-label to treat a variety of hyperkinetic movement disorders in adults and children.

Valbenazine, a selective VMAT2 inhibitor, is metabolized to an isomer of alpha-dihydrotetrabenazine after the amino acid valine is cleaved. In comparison, deutetrabenazine, a deuterated version of tetrabenazine, and metabolized to four isomers targeting VMAT2 as well as serotonin and dopamine receptors. All three drugs have similar occupancy of VMAT2, but tetrabenazine and deutetrabenazine have a relatively shorter half-life with additional dosing options while valbenazine has a longer half-life and is administered only once per day [1,2].

Compared to adults, less is known about prescribing practices and patient experiences with VMAT2 inhibitors in the pediatric population. In 2006, a retrospective review of tetrabenazine use as second line therapy for movement disorders in 31 children found that tetrabenazine was effective as a second line agent, and that children often required higher doses than adults [23]. A 2012 systematic review of tetrabenazine use in movement disorders found it was effective and tolerable in children and adults, with side effects (depression, fatigue, parkinsonism, somnolence) related to increased dose and age of patients [24]. Building on this, our findings offer information regarding common indications, dosages, efficacy, side effects, and barriers to treatment for pediatric patients at a large pediatric movement disorders center, thus providing further guidance for providers utilizing VMAT2 inhibitors to treat pediatric movement disorders.

## Methods

We performed a retrospective chart review of patients treated with VMAT2 inhibitors at a large pediatric movement disorders center from 2011 to 2023 by a pediatric movement disorders specialist at Texas Children’s Hospital. Demographics, medical history, and clinical notes were reviewed for all patients. Information regarding diagnosis and indication for VMAT2 prescription, dosing, response, side effects, barriers to medication access, and efficacy was collected. All patients had at least one clinical follow up, although the follow up timing varied based on the individual provider’s recommendations. Efficacy was determined by patient/physician report as documented within the clinical notes and Clinical Global Impression-Improvement (CGI-I) scale based on the patient/parent report and examiner’s impression on clinical examination (Table 1) and derived from the clinical notes [25]. Meaningful improvement was defined as CGI-I of 1–2.

**Table 1:** Clinical Global Impression-Improvement (CGI-I) scale. To calculate the CGI-I Scale, the clinician compares the patient’s overall clinical condition to the one-week period just prior to the initiation of medication use, known as the baseline visit. The CGI score is first documented at this baseline visit, and for CGI-I, the comparison above is considered [25].


SCORE	INTERPRETATION

1	Very much improved

2	Much improved

3	Minimally improved

4	No change from baseline (initiation of treatment)

5	Minimally worse

6	Much worse

7	Very much worse since the initiation of treatment


## Results

### Prescribing Practices

From 2011 to 2023, 340 patients (65.3% male) were prescribed at least one VMAT2 inhibitor at our pediatric movement disorders clinic. The patients had an average age of 11.9 years and median age of 12 years and range of 1.7 to 19 years (Table 2). Of these 340 patients, the treatment indication for each patient was tics in 265 (77.26%), 30 (8.75%) non-HD chorea, 27 (7.87%) stereotypy, 22 (6.41%) dystonia, 4 tardive dyskinesia (1.17%), 1 akathisia (0.29%) and 1 myoclonus (0.29%). Nine patients had more than one indication for treatment.

**Table 2:** Population demographics of children prescribed VMAT2 inhibitors, and number of unique prescriptions.


	TETRABENAZINE	DEUTETRABENAZINE	VALBENAZINE

Number of patients (N)	334	20	5

Mean Age (Years)	11.8	13.4	15

Sex			

Male	217	11	5

Female	117	9	0

Indication			

Tics	260	12	4

Chorea	30	3	1

Stereotypy	27	1	0

Dystonia	21	1	0

Tardive dyskinesia	3	1	0

Akathisia	1	0	0

Myoclonus	1	1	0


Of the 340 patients in our study, we found 359 unique prescriptions for VMAT2 inhibitors; several patients (N = 19) received prescriptions for more than one VMAT2 inhibitor. Most prescriptions (N = 334, 93%) were tetrabenazine, with a few (N = 20, 5.6%) prescribed deutetrabenazine and even less (N = 5, 1.4%) valbenazine (Table 2).

**Figure 1:**
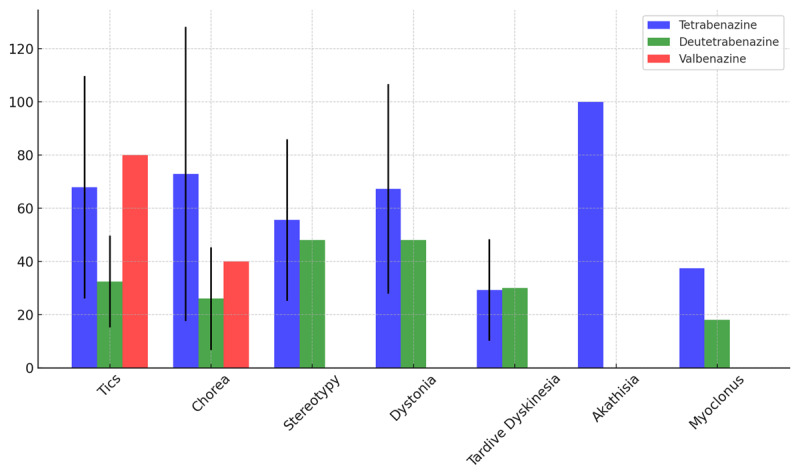
Average total mg/day dosing of VMAT2 inhibitors (black bar indicating standard deviation).

The average VMAT2 inhibitor doses varied depending on indication and which VMAT2 inhibitor was prescribed (Figure 1). Of the 334 patients with prescriptions for tetrabenazine, 78 (23.4%) were never started, most commonly due to insurance denials. Tetrabenazine was primarily prescribed for tics (76.9%), followed by chorea (8.7%), dystonia (6.3%) and stereotypy (7.2%), with less than 1% of prescriptions for tardive dyskinesia, akathisia or myoclonus (Table 2). The average daily dosing was 68.8 mg with a standard deviation of 42.8 mg, median of 56.25 mg and range of 6.25 to 225 mg. Although we typically use standardized dosing, final mg/kg/day total dosing averaged 1.5 (±1.3) mg/kg/day within this cohort.

Of the 20 patients with deutetrabenazine prescriptions, 5 (25%) were never started. Like tetrabenazine, deutetrabenazine was primarily prescribed for tics (65%), followed by chorea (15%), dystonia (5%) stereotypy (5%), tardive dyskinesia (5%) and myoclonus (5%) (Table 2). The average daily dosing was 30.7 mg with a standard deviation of 15.1 mg, median of 24 mg and range of 18 to 54 mg. Average weight-based dosing was 0.5 (±0.3) mg/kg/day.

There were 5 prescriptions for valbenazine; 1 (20%) was never started. Valbenazine was primarily used for treatment of tics (80%) or chorea (20%). The average daily dosing was 60 mg with a standard deviation of 23.1 mg, median of 60 and range of 40 to 80 mg. Average weight-based dosing was 1.3 (±0.4) mg/kg/day.

### Efficacy

**Figure 2:**
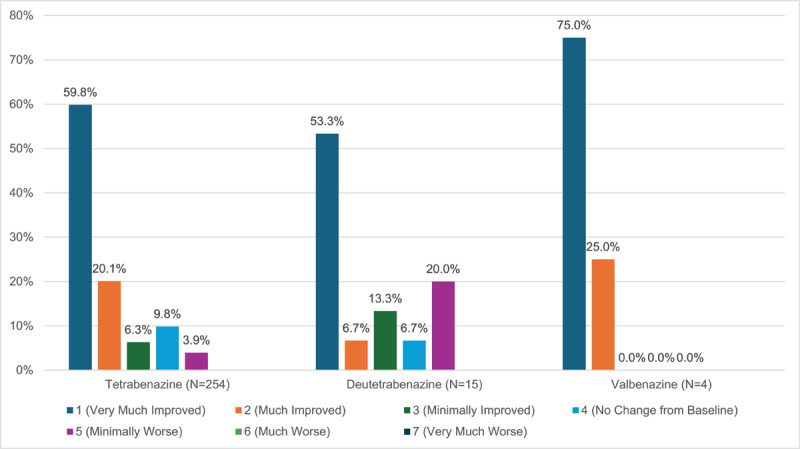
Percentage of patients undergoing treatment with VMAT2 inhibitor with resultant Clinical Global Impression-Improvement.

Of the 359 total prescriptions for VMAT2 inhibitors, 75.8% (N = 275) successfully commenced treatment. Within this group, the majority (62.8%) reported clinical improvement with an average Clinical Global Impression-Improvement (CGI-I) of 1.8 (±1.2); some (11.5%, N = 40) reported no improvement. The CGI-I score range was 1 to 5 among this patient population (Figure 2). Meaningful improvement was defined as CGI-I of 1–2. For the treatment of tics, 84% (N = 161) of patients subjectively reported meaningful reduction in tics; 93.3% (N = 28) of patients with chorea and 84.2% (N = 16) of patients with dystonia reported meaningful reduction of dystonia. 73.6% (N = 14) of patients with stereotypy and 75% (N = 3) of those with tardive dyskinesia reported meaningful reduction in dyskinesia. The average CGI-I for tics was 1.82 (±1.25) while the average CGI-I amongst VMAT2 inhibitors used for other conditions were chorea 1.65 (±0.88), dystonia 1.84 (±1.12), stereotypy 2.05 (±1.27) and tardive dyskinesia 2 (±2). Only one patient was prescribed tetrabenazine for akathisia with a CGI score of 2. Only one patient was prescribed VMAT2 inhibitor for myoclonus (prescribed tetrabenazine and then transitioned to deutetrabenazine) with CGI scores of 1.

**Figure 3:**
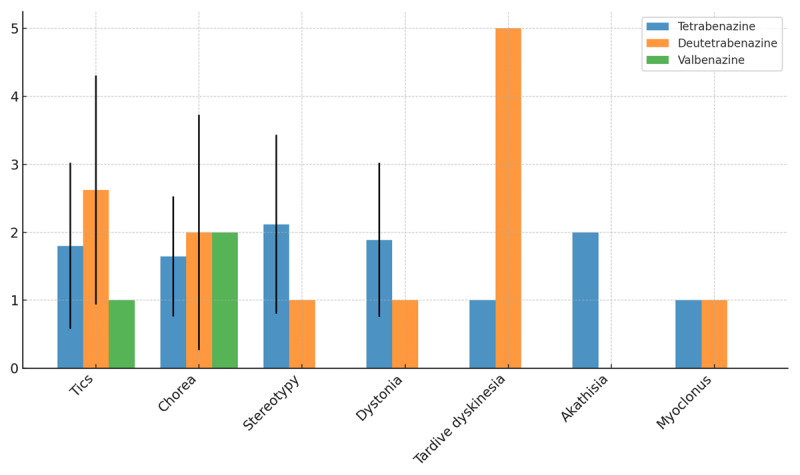
Average CGI-I by indication (black bar indicating standard deviation). Calculation of CGI-I score is outlined within the methods with lower scores indicating increased clinical improvement.

Of all patients who initiated treatment with tetrabenazine, 80.7% of patients (N = 205) subjectively reported symptomatic improvement whereas 14.17% had no improvement or worsening symptoms. The average CGI-I for tetrabenazine was 1.79 (±1.17), comparable to the average across the VMAT2 inhibitors. The CGI-I score ranged from 1 to 5 for tetrabenazine (Figure 2). For the treatment of tics, average CGI-I was 1.79 (±1.23) and 82.6% of patients had clinical benefit. The CGI-Is for treatment of chorea and dystonia were 1.6 (±0.81) and 1.89 (±1.13) respectively. For the treatment of tardive dyskinesia, CGI-I was 1 (Figure 3).

Of patients who started deutetrabenazine, 57.8% (N = 11) showed clinical improvement with an average CGI-I score of 2.33 (±1.68); CGI-I scores ranged from 1 to 5. For the treatment of tics, the average CGI-I was 2.63 (±1.68) and 75% of patients reported meaningful reduction of symptoms. The average CGI-Is for the treatment of chorea and dystonia were 2 (±1.73) and 1 respectively. For treatment of tardive dyskinesia, CGI-I was 5 (Figure 3).

All patients prescribed valbenazine who took the medication (N = 4) reported meaningful reduction in symptoms with an average CGI-I of 1.25 (±0.5). The highest CGI-I score in this group was a 2, demonstrating that there were no poor outcomes among the patients taking valbenazine. However, these results are limited by the low numbers of total prescriptions (4) and the resulting small range of CGI-I scores from 1 to 2. For the treatment of chorea, CGI-I was 2; for treatment of dystonia, CGI-I was 1 (Figure 3).

### Side Effects

Overall, 63.9% of patients reported side effects, most commonly drowsiness/somnolence. However, only 35 (10.1%) necessitated discontinuation due to side effects. About half of side effects (49.54%) were considered central nervous system (CNS) side effects, including somnolence, increased seizures, cognitive side effects and dizziness. Of the CNS effects, most patients (N = 99, 91.7%) experienced somnolence/drowsiness (Table 3). Of note, we categorized movement disorders side effects separately from the general CNS side effects category. 27 patients that experienced side effects had resolution of side effects with dose reduction.

**Table 3:** Adverse effects reported by patients taking VMAT2 inhibitors.


CATEGORY	ADVERSE EFFECT	ALL VMAT2	TETRABENAZINE	DEUTETRABENAZINE	VALBENAZINE

**None**	None	125 (36%)	114 (44.5%)	8 (53.3%)	3 (75%)

**Central Nervous System**	Drowsiness/Somnolence	99 (45.4%)	95 (37.1%)	4 (26.7%)	0 (0%)

	Increased Seizures	3 (1.4%)	3 (1.2%)	0 (0%)	0 (0%)

	Cognitive Side Effects	2 (0.9%)	2 (0.8%)	0 (0%)	0 (0%)

	Dizziness	4 (1.8%)	4 (1.6%)	0 (0%)	0 (0%)

**Movement Disorders**	Akathisia	5 (2.3%)	5 (2%)	0 (0%)	0 (0%)

	Neuroleptic Malignant Syndrome*	1 (0.5%)	1 (0.4%)	0 (0%)	0 (0%)

	Acute Dystonic Reactions*	15 (6.9%)	13 (5.1%)	1 (6.7%)	1 (25%)

	Parkinsonism	5 (2.3%)	5 (2%)	0 (0%)	0 (0%)

	Tremor	1 (0.5%)	1 (0.4%)	0 (0%)	0 (0%)

	Worsening of underlying symptom	6 (2.8%)	4 (1.6%)	2 (13.3%)	0 (0%)

**Gastrointestinal**	Nausea/Vomiting	7 (3.2%)	7 (2.7%)	0 (0%)	0 (0%)

	Diarrhea	2 (0.9%)	2 (0.8%)	0 (0%)	0 (0%)

	Dysphagia	1 (0.5%)	1 (0.4%)	0 (0%)	0 (0%)

	Weight Gain	5 (2.3%)	5 (2%)	0 (0%)	0 (0%)

**Psychiatric**	Suicidal Ideation	3 (1.4%)	3 (1.2%)	0 (0%)	0 (0%)

	Anger/Agitation	8 (3.7%)	8 (3.1%)	0 (0%)	0 (0%)

	Depression	17 (7.8%)	16 (6.3%)	1 (6.7%)	0 (0%)

	Anxiety	9 (4.1%)	8 (3.1%)	1 (6.7%)	0 (0%)

	Mood Instability	8 (3.7%)	8 (3.1%)	0 (0%)	0 (0%)

	Insomnia	1 (0.5%)	1 (0.4%)	0 (0%)	0 (0%)

	Nightmares	1 (0.5%)	1 (0.4%)	0 (0%)	0 (0%)

**Cardiovascular**	Palpitations	2 (0.9%)	2 (0.8%)	0 (0%)	0 (0%)

	Bradycardia	2 (0.9%)	1 (0.4%)	0 (0%)	0 (0%)

**Other**	Irregular menses	1 (0.5%)	1 (0.4%)	0 (0%)	0 (0%)

	Sweating	1 (0.5%)	1 (0.4%)	0 (0%)	0 (0%)

	Fever	1 (0.5%)	1 (0.4%)	0 (0%)	0 (0%)

	Rash	1 (0.5%)	1 (0.4%)	0 (0%)	0 (0%)

	Blurry Vision	1 (0.5%)	1 (0.4%)	0 (0%)	0 (0%)


*Patients were concurrently taking dopamine receptor blocking medication(s).

Of patients prescribed tetrabenazine, 81 (31.89%) discontinued the medication. Of these, 24 (29.63%) were discontinued due to cost of medication, and 32 (39.51%) were due to side effects. Seven patients who discontinued tetrabenazine due to side effects successfully transitioned to deutetrabenazine with resolution of the bothersome side effects, and one patient similarly transitioned to valbenazine with similar resolution of side effects. Of the remaining patients discontinued from tetrabenazine, 15 patients (18.52%) cited medication insufficiency, one had medication interactions, and one had medication delivery issues. Five patients (35.71%) who were prescribed deutetrabenazine were discontinued, citing inefficacy (2 patients), side effects (2 patients) and resolution of the underlying symptom to be treated (1 patient). Of the 4 patients prescribed valbenazine, one (20%) was discontinued due to side effects.

### Barriers to access

Of the 359 total prescriptions, 194 (59.3%) experienced at least one denial from insurance companies. Financial concerns regarding medication affordability were common amongst patients with 65 (18.2%) patients reporting. There were 27 (7.5%) initial denials due to the requirement *CYP2D6* genetic testing either by insurance or by the dispensing pharmacy. Reasons for insurance denial included “Lack of FDA Approval” and “Age under 18”.

147 of 334 (44%) patients prescribed tetrabenazine were originally approved and 98 of 187 (52.4%) received subsequent approval after appeal. Furthermore, 12 of 334 (3.6%) patients were denied coverage for increased dose requests. 62 of 334 (18.6%) patients felt the medication was ultimately cost prohibitive. There were delivery issues with 15 of 334 (4.5%) patients and other pharmacy barriers for 32 of 334 (9.6%) patients. Pharmacies specifically required *CYP2D6* testing for 29 of 334 (8.7%) patients and a letter of medical necessity for 3 of 334 (1%) patients.

10 of 20 (50%) patients prescribed deutetrabenazine were originally approved, and 2 of 20 (10%) subsequently approved on appeal. 2 of 20 (10%) patients felt the medication was ultimately cost prohibitive. Of note, all patients apart from one, had a prior VMAT2 inhibitor trial or prescription.

2 of 5 (40%) patients prescribed valbenazine were originally approved by insurance while 2 of 3 (66.7%) were subsequently approved on appeal. One patient believed the medication to be cost prohibitive. Similarly, only one patient was prescribed valbenazine prior to any other prescriptions/trials of other VMAT2 inhibitors.

*CYP2D6* genotype testing was completed in 27 patients to evaluate their metabolism of VMAT2 inhibitor medications. Sixteen (59.2%) were normal or intermediate to normal metabolizers. The remaining patients were extensive metabolizers (N = 3), extensive to intermediate metabolizers (N = 3), intermediate metabolizers (N = 2) poor metabolizers (N = 2), or intermediate to slow metabolizers (N = 1). *CYP2D6* testing results did not correlate with dose, clinical efficacy, nor side effects.

## Discussion

RCTs have shown that tetrabenazine and deutetrabenazine are safe and effective for treating tardive dyskinesia [10,11,12,13]; valbenazine is also effective for patients with chorea in HD and approved for this indication [14]. While long-term experience and many open-label studies have documented safety and efficacy of VMAT2 in the treatment of associated with Tourette syndrome (TS) [15,16,17], these benefits could not be statistically proven in RCTs. Two RCTs in pediatric patients with TS showed that deutetrabenazine was well tolerated but did not significantly reduce tic severity [16,17]. There are many possible explanations for these negative outcomes, including lack of sensitivity of the Yale Global Tic Severity Scale (YGTSS), which is the main outcome measure in most clinical trials, marked placebo and nocebo response in TS [18], and underdosing [19]. Ongoing studies are exploring the efficacy and tolerability of valbenazine for pediatric patients with dyskinesia due to cerebral palsy [20].

In 2019, Niemann and Jankovic published real-world experiences with VMAT2 inhibitors, primarily used to treat HD-related chorea and tardive dyskinesia [21]. In children, VMAT2 inhibitors are often prescribed for tics, chorea or dystonia. However, adult, and especially pediatric, patients may struggle to access VMAT2 inhibitors due to insurance and financial barriers, lack of FDA approval, and potential side effects or medication interactions. Common side effects include sedation, insomnia, headache, weight gain, akathisia, agitation and nausea [9,16]. Rare side events include depression, suicidality, QT prolongation, and neuroleptic malignant syndrome. Although potential for suicidality has been often cited as a reason to avoid VMAT2 inhibitors and these drug labels contain “black box” warnings of depressed mood and suicidal ideation, several studies found that tetrabenazine use was not associated with an increased incidence of depression or suicidality [22].

Generally, VMAT2 inhibitors are the preferred treatment for several hyperkinetic movement disorders, especially in adults. These include chorea associated with HD and tardive dyskinesia as VMAT2 inhibitors are often more effective than the dopamine receptor blocking medications and are not associated with metabolic syndrome nor cause tardive dyskinesia [26]. Because VMAT2 inhibitors are not yet approved for the treatment of tics associated with TS, these drugs are not frequently prescribed globally for this indication, although we have had a long-term experience with these dopamine depleters at Baylor College of Medicine [19,21]. As these patients were only followed at our pediatric movement disorders center, none of these were included in the prior publications.

Our study suggests that VMAT2 inhibitors are generally effective and safe for treating pediatric hyperkinetic movement disorders. While side effects were common, most were mild and seven patients were transitioned from tetrabenazine to deutetrabenazine with resolution of side effects, and one to valbenazine with similar effect. This study provides insight into prescribing practices, the limited use of *CYP2D6* testing and barriers to pediatric access from our large pediatric movement disorders center.

Prescribing practices showed that the vast majority of VMAT2 inhibitor prescriptions were for tetrabenazine, consistent with the widespread use of this medication and its status as the original medication in this class. While the number of deutetrabenazine and valbenazine prescriptions were considerably lower compared to tetrabenazine, all three medications were used for similar indications: most often tics, followed by chorea. Few patients were also prescribed tetrabenazine or deutetrabenazine for stereotypy, myoclonus and tardive dyskinesia.

The best clinical improvement based on CGI-I scores were seen in valbenazine followed by tetrabenazine and then deutetrabenazine, but without well-designed, head-to-head comparison trials there is no evidence-based data on the relative efficacy and safety of the three VMAT2 inhibitors. Our real-world experience, however, provides insights into the use of these drugs in pediatric hyperkinetic movement disorders. It also highlights the heterogeneity of response and the importance of trying different VMAT2 inhibitors and optimizing the dosages, specifically tailored to the needs of individual patients.

CGI-I scores suggest VMAT2 inhibitors were most effective for chorea, followed by tics and dystonia within this pediatric cohort. Their efficacy in treating hyperkinetic movement disorders is theorized to stem from reduction of excessive dopaminergic signaling within the brain. Unlike dopamine receptor blocking agents which block dopamine receptors, VMAT2 inhibitors act by depleting the presynaptic storage of dopamine and as such have not been associated with tardive dyskinesia, although they can cause transient acute dystonic reaction, akathisia, and drug-induced parkinsonism [27].

The safety and efficacy of VMAT2 inhibitors are largely dependent on dosing. This is especially true in the pediatric population. Tetrabenazine was commonly started at 6.25 mg/day followed by slow titration of 12.5 mg/week to a maximum dose (in this cohort) of 225 mg/day divided in three doses. Deutetrabenazine was initiated in 6 mg twice daily, with increases of 6–12 mg/week to a maximum daily dose of 54 mg/day divided twice a day. There were no patients within this cohort that received the extended-release formulation which would be dosed once daily [28]. Lastly, valbenazine was initiated at 40 mg/day and then increased to 80 mg/day in one week, continuing the lowest effective dose.

Patients with chorea and dystonia required the highest doses to achieve the most optimal clinical improvement. In general, however, we [19] and others have observed that higher doses are necessary to effectively treat pediatric hyperkinetic movement disorders compared to adult population. Indeed, Jain et al. [23] reported that dosages of tetrabenazine as high as 350 mg per day were required to treat their children with hyperkinetic movement disorders; the highest dose of tetrabenazine in our cohort was 225 mg per day. Further research is needed to determine optimal pediatric dosing, particularly for deutetrabenazine and valbenazine.

Our data also suggests that VMAT2 inhibitors are well tolerated with somnolence as the most common side effect; only 10.1% of patients necessitated discontinuation of the drug due to side effects. Similar side effect profiles were seen across tetrabenazine and deutetrabenazine prescriptions; our sample size for valbenazine was too small to observe these trends.

Rare serious side effects included suicidal ideation in 3 patients taking tetrabenazine. Of note, a black box warning regarding depression and suicidal ideation is issued for tetrabenazine, deutetrabenazine and valbenazine. In our cohort of patients, acute dystonic reaction and neuroleptic malignant syndrome were observed, but these patients were also taking another dopamine receptor blocking medication which could have contributed to these side effects [29,30]. However, the simultaneous use of antidepressants or other dopamine receptor blocking agents is not a contraindication to treatment with a VMAT2 inhibitor [31,32]. All bothersome side effects resolved with reduction of the dose, discontinuation of medication, or transitioning from tetrabenazine to either deutetrabenazine or valbenazine.

Insurance denials affected all three VMAT2 inhibitors at similar rates: 23.4% for tetrabenazine, 25% for deutetrabenazine, and 20% for valbenazine. This suggests that barriers to medication initiation may be similar across all three options. However, given patients were often prescribed tetrabenazine first with plans of transitioning to either deutetrabenazine or valbenazine due to side effects, similar rates of denials for these better tolerated agents can be quite impactful for patients. Though some appealed successfully, others were denied multiple times. About 1 in 5 patients expressed concerns about medication affordability, and a smaller fraction was denied due to the need for *CYP2D6* genetic testing. Some patients experienced significant barriers and delays due to this requirement of testing while also denying the request for the genetic testing, often frustrating patients and clinicians.

Denial rates suggest insurers view all three medications similarly with none being approved more frequently, again with the caveat of small sample sizes of deutetrabenazine and valbenazine as well as these often being prescribed after a failure of tetrabenazine. As a result, many providers will first prescribe tetrabenazine in children as deutetrabenazine and valbenazine are often not approved unless tetrabenazine has already been used. Such insurance denials are unfortunately common given the lack of FDA approval in pediatric patients. Despite these challenges, the order of prescriptions may help minimize these denials. Providers should be proactive and transparent with families when prescribing VMAT2 inhibitors to pediatric patients, recognizing that there may be delays in accessing the medication and informing the family about potential insurance and financial barriers.

*CYP2D6* metabolizer profiles ranged among our population, however, did not correlate with dosing changes, clinical efficacy, nor side effects. In theory, patients who are slower metabolizers will require lower doses of medication to be effective; extensive metabolizers may need higher doses. However, our findings suggest that, despite insurance-mandated testing, it may have limited clinical utility within the pediatric population within the context of VMAT2 inhibitor treatment. Although genetic testing may potentially identify individuals who are slow metabolizers and are, therefore, more likely to develop side effects, we generally rely on the clinical response in individual patients in monitoring for potential side effects, rather than genetic testing.

This study, conducted at a large urban medical center, may not generalize to all populations. Data were obtained from clinical notes, so may have missed cases lost to follow-up or treated elsewhere. Objective rating scales were not routinely or consistently used by clinicians; therefore, aside from the Clinical Global Impression–Improvement (CGI-I) scale, we lack standardized objective outcome measures. We also recognize that our relatively small sample size of deutetrabenazine and valbenazine prescriptions may limit conclusions. Furthermore, our center is a specialized pediatric movement disorders center, patients are often pre-selected for evaluation within these clinics with a chief complaint of abnormal movements and often failed other treatments provided by general neurologists, or symptoms may be considered severe.

Nonetheless, more RCTs are needed to include the pediatric population to expand the on-label indications for children. As noted earlier, there are several challenges to designing RCTs for TS, and tics are the most common indication for VMAT2 inhibitor treatment. Future studies should consider use of adjunctive outcome measures such as patient-caregiver reported outcomes and video-based tic-assessments which may provide more robust and clinically meaningful endpoints. Furthermore, a dose optimization phase should be considered to help identify the most effective and tolerable dose for each participant which would minimize the risk of underdosing seen in prior trials. Lastly, one could consider an initial open label phase where all patients receive treatment and subsequently randomized in a double-blind manner to either continue treatment or switch to placebo. However, designing RCTs for other pediatric hyperkinetic movement disorders such as chorea and dystonia presents unique difficulties. These conditions are rare, heterogenous in etiology (e.g. genetic, metabolic, autoimmune), and often require individualized treatment approaches making standardized trial protocols and uniform outcome measures particularly challenging. These complexities underscore the need for alternative study designs and multicenter collaborations to achieve adequately powered trials.

Our findings support the clinical utility of VMAT2 inhibitors as effective and generally well-tolerated treatments for pediatric hyperkinetic movement disorders. Although side effects were relatively common, they were typically mild and manageable through dose adjustments or switching to newer agents such as valbenazine or deutetrabenazine. These observations suggest that personalized dosing strategies and careful medication selection can optimize tolerability in pediatric patients. Importantly, we identified substantial barriers to access, particularly related to insurance denials and high out-of-pocket costs, which may delay or limit appropriate treatment. Although some insurers required CYP2D6 genotyping, test results did not appear to influence clinical decisions or outcomes, calling into question the utility of routine pharmacogenetic testing in this context. These findings highlight the need for more standardized treatment protocols and equitable access to VMAT2 inhibitors, as well as further research to establish clear clinical guidelines tailored to the pediatric population.

## Financial Disclosures for the previous 12 months

MH served on a pediatric advisory board for Abbvie. The institution of MH has received research support from the NIH, Emalex, and Neurocrine Biosciences for clinical trials research and unrelated to this study.
